# Assessing the Impact of Pre-Soaking to Enhance Laundering Efficacy of Firefighter Turnout Gear

**DOI:** 10.3390/toxics12080544

**Published:** 2024-07-27

**Authors:** Md Tanjim Hossain, R. Bryan Ormond

**Affiliations:** Textile Protection and Comfort Center (TPACC), Wilson College of Textiles, North Carolina State University, Raleigh, NC 27606, USA; mhossai9@ncsu.edu

**Keywords:** firefighters, turnout gear, PAHs, PAH desorption, presoaking, cleaning efficacy

## Abstract

Firefighters are exposed to hazardous chemicals at fire scenes, including polycyclic aromatic hydrocarbons (PAHs) among many others, which pose significant health risks. Current laundering practices are ineffective at removing persistent contaminants from turnout gear, necessitating further research to optimize cleaning methods. This study explores the impact of presoaking prior to the laundering process and the factors that can affect its effectiveness, including the presoaking duration and detergent concentration, in PAH removal when laundering. For this, contaminated fabric swatches were subjected to various presoaking durations (1, 3, and 12 h) and detergent concentrations (99:1 and 90:10 water-to-detergent ratios) before undergoing bench-scale washing. The cleaning efficacy was assessed for 16 PAH compounds, including both low-molecular-weight (LMW) PAHs and high-molecular-weight (HMW) PAHs. Moreover, the removal mechanisms of PAHs from turnout gear were fundamentally explained using partition coefficients and standard affinities with different parameters during washing. The results demonstrate that 3 h and 12 h of presoaking lead to 2.8 and 4.3 times greater HMW PAH removal, respectively. After 12 h of presoaking in a 90:10 water-to-detergent ratio, 97% of the LMW PAHs and 78% of the HMW PAHs were removed, compared to only an 11% removal of the HMW PAHs with a 99:1 ratio. Additionally, direct washing with a 90:10 ratio achieved comparable efficacy to that of presoaking with the same water-to-detergent ratio, indicating the crucial role of detergent concentration during laundering. These findings offer valuable insights for optimizing firefighter safety practices, emphasizing the role of presoaking and the appropriate methods to perform presoaking to mitigate firefighters’ occupational exposure risks to toxic substances and ensure gear reliability.

## 1. Introduction

Firefighting is one of the most hazardous occupations, characterized by high rates of fatal injuries, skin burns, and heat-stress-related casualties [1,2]. In addition to heat-related injuries, firefighters face significant health hazards due to chemical exposure, including an elevated risk of developing specific types of cancer, such as lung, bladder, prostate, melanoma, and colorectal cancers [3,4,5,6,7]. Therefore, the International Agency for Research on Cancer (IARC), an agency of the World Health Organization (WHO), classified the firefighting occupation as “carcinogenic to humans” (Group 1) with sufficient evidence for bladder cancer and mesothelioma [8].

Firefighters are exposed to a wide range of carcinogenic compounds at fire scenes. Volatile organic compounds and polycyclic aromatic hydrocarbons (PAHs) are prevalent in smoke, and chronic exposure to these substances can lead to serious health issues, including cancer [9,10,11,12,13,14]. PAH compounds can be classified into two groups: low-molecular-weight (LMW) PAHs and high-molecular-weight (HMW) PAHs [15]. LMW PAHs contain two or three aromatic rings in their structure, whereas HMW PAHs contain four to six aromatic rings. These compounds can accumulate on firefighters’ gear during firefighting activities. For instance, studies by the Queensland (AUS) Fire and Rescue Service Scientific Branch have detected PAHs both inside and outside of turnout gear, with significantly lower levels inside the ensemble. Additionally, Kirk et al. measured PAH concentrations on the turnout gear of instructors involved in structural live-fire training sessions and found that the total concentration of PAHs on the outer surface of the gear ranged from 69 to 290 ng/cm^2^ [10]. 

Skin can act as a primary route of entry for PAHs or other hazardous substances generated at fire scenes assuming the inhalation route is protected through the proper and continuous use of respiratory protection [16,17,18]. Dermal exposure can be a significant pathway for firefighters to absorb these contaminants, as the skin can be permeable to toxic substances [19,20]. If turnout gear is not cleaned properly, contaminants can transfer from the gear to the skin, increasing the risk of their absorption into the body. Therefore, firefighters must decontaminate themselves and their turnout gear after participating in fire suppression to minimize the risk of exposure to hazardous compounds. The NFPA 1851: Standard on Selection, Care, and Maintenance of Protective Ensembles for Structural Fire Fighting and Proximity Fire Fighting provides guidelines to decontaminate turnout gear [21]. This standard recommends two types of cleaning: routine cleaning/on-scene decontamination and advanced cleaning/laundering.

The NFPA recommends performing advanced cleaning such as laundering at least twice per year, although there is a significant push for gear to be laundered after every instance of fire exposure. Besides laundering, some dry-cleaning methods like ozone cleaning and liquid carbon dioxide (CO_2_) cleaning are used in the firefighting industry. However, these methods are not yet widely available due to their limitations, unavailability, or limited research on their effect on PPE performance. For example, ozone cleaning has been shown to generate new oxygenated PAH compounds that could be more harmful than the original PAHs [22]. CO_2_ cleaning has demonstrated effectiveness in removing semi-volatile organic compounds (SVOCs); however, the lack of mechanical action in this decontamination process limits its effectiveness in removing particulate contamination generated from smoke and soot at fire scenes [23]. Therefore, the NFPA 1851-approved aqueous washing method/laundering is the most widely used washing method in the firefighting industry. However, previous studies have indicated that laundering is insufficient at removing larger-molecular-weight hydrophobic organic compounds like PAHs from firefighter gear [24,25,26,27]. In some cases, the PAH concentrations were found to be almost unchanged before and after laundering, suggesting that current laundering systems do not effectively remove these compounds. For instance, Banks et al. assessed the effect of laundering on SVOCs, including PAHs, and found limited success in reducing the concentration of certain PAH compounds from contaminated gear [28]. They concluded that current laundering systems are ineffective against semi-volatile organic compounds. Abrard et al. measured the concentration of benzo[a]pyrene (BaP), a Group 1 carcinogen, on the outer surface of a firefighter’s jacket after participating in a single training session, finding levels of 113.75 ± 45.03 μg/m^2^ [24]. They performed decontamination using a non-ionic surfactant and concluded that the PPE cleaning procedure used was ineffective at reducing chemical contaminants like BaP. Recent research by Wilkinson et al. further demonstrated that machine laundering and preliminary exposure reduction (PER) techniques are ineffective at removing semi-volatile PAHs embedded deep within the fibers of bulky PPE [29]. Although they reported significant removal of PAHs from the exterior of turnout gear, they found limited success in removing PAHs from inner layers. Keir et al. reported that laundering the PPE of firefighters who participated in emergency fire suppression removed 61% of PAHs on average, although they did not specify the removal efficacy for individual PAHs detected on the PPE [26]. Many of these studies used wipes to collect PAHs from the outer surface of fabrics to calculate the cleaning efficacy. However, wipes are unable to collect the total amount of PAHs from the turnout gear fabrics, and in some cases, the effectiveness of wipes in collecting PAHs from ensembles was unknown.

Exposure to contaminants at fire scenes is not uniform, and turnout gear among firefighting personnel often shows varied levels of contamination. To ensure uniform contamination for cleaning validation, the NFPA suggests applying a known concentration of liquid contamination before performing washing [21]. Forester et al. applied a known concentration of PAH compounds to the outer-shell fabrics of turnout gear following the NFPA cleaning validation process to test the removal efficacy of PAHs during laundering. After the contamination, the fabrics were attached to turnout gear jackets and laundered to investigate the removal efficacy against these persistent PAH compounds. Forester et al. reported 18% and 12% removal rates of chrysene and benzo(a)pyrene, respectively, after laundering gear with a commonly used detergent in the firefighting industry [30]. Hossain et al. demonstrated that removal efficacy decreases with increasing molecular weight of PAH compounds, with the lowest removal efficacy observed for PAHs containing five or six rings in their structure [31]. Therefore, it can be assumed that compounds like indeno[1,2,3-cd]pyrene (Ind) and benzo[g,h,i]perylene (B[ghi]P), which have six aromatic rings, would exhibit much a lower removal efficacy if firefighters’ gear is exposed to these compounds. These findings demonstrate that the current laundering practice is inefficient in removing PAHs, especially those with a high molecular weight.

Since current laundering practices fail to remove persistent chemical contaminants, it is crucial to conduct further research to identify the most effective laundering methods to ensure the health and safety of firefighters. Presoaking contaminated turnout gear could enhance cleaning performance. The NFPA 1851 standard permits presoaking the outer-shell layer of the gear as a pretreatment before laundering. However, the standard does not provide specific guidelines for presoaking, leaving the process to the recommendations of cleaning product manufacturers [21]. Many commercially available detergents used by firefighters recommend presoaking to achieve optimal cleaning results against persistent contaminants. Although these manufacturers typically provide instructions on the detergent-to-water ratio for presoaking, neither the NFPA nor the manufacturers offer guidelines on the required presoaking duration. Although firefighters occasionally presoak their heavily contaminated gear before washing it, there is limited information on how presoaking affects cleaning performance due to the lack of any published research in this area. Since laundering alone has proven ineffective in removing persistent HMW PAHs, comprehensive research is needed to determine whether presoaking can improve the cleaning performance of laundering and identify major factors affecting removal performance, such as detergent concentration and presoaking duration. The removal or desorption of PAHs from contaminated fabrics through the laundering or washing process is essentially the reverse of the adsorption process (such as a dyeing process), and the thermodynamic and kinetic parameters impact the removal of PAHs from the fabrics [32,33]. Therefore, it is important to calculate the partition coefficient (K) and standard affinity (Δμ^θ^) between the fabric and solution under different washing conditions to understand the PAH desorption process in fabrics during washing.

The primary objective of this research is to evaluate the effect of presoaking in removing a wide range of semi-volatile chemical contaminants from turnout gear and improve the effectiveness of the laundering process. For this, outer-shell fabric swatches were contaminated using a standard reference mixture of 16 PAHs, and a popular non-ionic detergent was used for washing these. Initially, a full-scale wash was performed in a washer extractor without presoaking to assess the performance of laundering alone against the 16 PAHs. Subsequently, a bench-scale washing process was employed to simulate laundering with and without presoaking for a comparative analysis. Various presoaking durations were tested to determine the optimal presoaking time for achieving the best cleaning performance. Additionally, the effect of different detergent concentrations during presoaking was analyzed. Washing the swatches with a high detergent concentration without presoaking was also conducted to evaluate whether the presoaking itself or the detergent concentration played a major role in cleaning efficacy. Moreover, key parameters for the PAH removal process, including the partition coefficient (K) and standard affinity (Δμ^θ^), were measured to explain how PAH removal is correlated with these parameters on a more fundamental level. This study specifically aims to evaluate the effectiveness of various presoaking durations and detergent concentrations in reducing PAH contamination in firefighter turnout gear. The findings from this study will provide valuable insights to firefighters, cleaning service providers, and firefighter standards committees, including the NFPA, on the importance and effectiveness of presoaking, thereby contributing to advancements in cleaning practices.

## 2. Materials and Methods

### 2.1. Material

#### 2.1.1. Fabric

For this study, a flame-resistant fabric commonly used in wildland firefighting ensembles was chosen as the contamination substrate for all cleaning experiments due to its availability. The Sigma™ fabric (Safety Components, St. Louis, MO, USA) was composed of 5% meta-aramid/17% polyamide/6% para-aramid/32% Lenzing^®^ FR and had a silicone-based water-repellent finish. This material is similar to many structural firefighter outer-shell materials and is certified to NFPA 1977, 1975, and 1951 standards. The weight of a (5 × 4 cm) fabric swatch was 0.5 g.

#### 2.1.2. Chemical Contaminants

A repeater pipette (Eppendorf, Hamburg, Germany) was used to dispense PAH droplets of the reference mix on the fabric swatch. A QTM PAH Standard Mix containing 16 PAH compounds in methylene chloride, shown in Table 1, was purchased from Sigma-Aldrich Inc., St. Louis, MO, USA (CRM47930). The concentration of each PAH compound in the stock solution was 2000 ng/μL. The standard mix was packaged in 2 mL amber vials and kept in a refrigerator at 4 °C. Dilutions of the standard mix were made for calibration of analytical instrumentation.

The 16 PAHs include seven LMW PAHs: naphthalene (Nap), acenaphthylene (Acy), 2-bromo naphthalene (2-Br), acenaphthene (Ace), fluorene (Fle), phenanthrene (PHE), and anthracene (An). The remaining nine are HMW PAHs: fluoranthene (Fla), pyrene (Py), benz[a]anthracene (B[a]A), chrysene (Chr), benzo[b]fluoranthene (B[b]F), benzo[a]pyrene (B[a]P), indeno[1,2,3-cd]pyrene (Ind), dibenz[a,h]anthracene (D[ah]A), and benzo[g,h,i]perylene (B[ghi]P).

#### 2.1.3. Detergent

One commercially available detergent that is commonly used in the firefighting industry was used for fabric decontamination during the washing process. This detergent contains a nonionic surfactant as one of its ingredients. According to its Safety Data Sheet (SDS), 6 oz of detergent is recommended for a 45 lb washing load. For presoaking, the detergent instructions suggest using a 90:10 water-to-detergent ratio for as long as required. After presoaking, laundering should be performed without adding any additional detergent to the washing machine.

### 2.2. Method

#### 2.2.1. Fabric Contamination

To perform full-scale washing in a washer extractor, a clean turnout jacket was used to which contaminated fabric swatches were attached. Fabric swatches (5 × 4 cm) were cut from the roll of fabric. Four hook-and-loop patches were stitched onto the jacket to hold contaminated fabric swatches for cleaning. Hook patches were stitched onto the jacket, and loop patches were stitched onto the fabric swatches. A repeater pipette (Eppendorf) was used to dispense six 5 µL droplets of the reference mix (60,000 ng of each PAH compound) on each fabric swatch after being stitched onto the loop patches, resulting in 3000 ng/cm^2^ of PAHs on each fabric swatch. After contamination, the fabric swatches were allowed to sit for 30 min at ambient conditions to let the contaminants penetrate the fabric surface and the solvent to evaporate. The swatches were then attached to the designated positions on the turnout gear using the hook-and-loop patches.

For the bench-scale washing, six 5 µL droplets of the reference mix (60,000 ng of each PAH compound) were similarly dispensed on the fabric swatch, resulting in 3000 ng/cm^2^ of PAHs on the fabric. Then, the fabric swatches were also kept for 30 min in ambient conditions before the bench-scale washing was performed.

#### 2.2.2. Full-Scale Washing without Presoaking

A UNIMAC^®^ 45 lb washer extractor, installed at Wilson College of Textiles, was used for laundering. Following the NFPA 1851 guidelines, laundering was performed at 40 °C (105 °F) for one hour without presoaking. During the washing cycle, 56.7 mL of detergent was used for a 14.4 lb load, scaled according to the manufacturer’s recommendations. After laundering, the samples were placed on a rack for 24 h to air dry. Four replicates were used for the full-scale washing.

#### 2.2.3. Bench-Scale Washing for Decontamination

**Figure 1:**
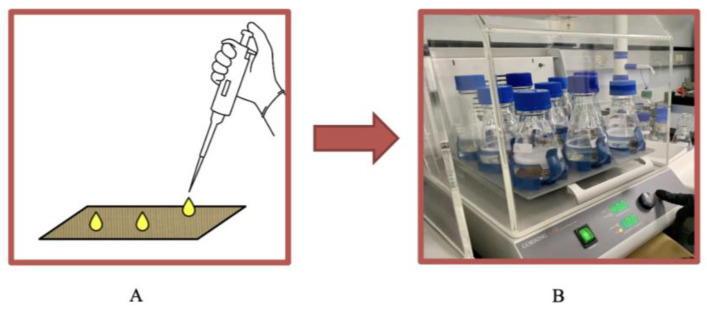
Contamination of turnout gear fabrics and bench-scale washing process; (**A**) Contamination of outer-shell fabrics using a repeater pipette; (**B**) Performing bench-scale washing in 250 mL Erlenmeyer flask placed in an LSE Corning^®^ bench-top shaking incubator.

A bench-scale washing method previously described by Girase et al. was used for the washing process in which contaminated swatches were washed in an incubated water shaker bath [34]. Contaminated swatches were transferred into 250 mL Erlenmeyer flasks containing water, detergent, and glass beads. Each flask contained a combined volume of 100 mL of water and detergent. To provide mechanical agitation during washing, 5 g of 4 mm glass beads were added to each flask. The flasks were then placed in an LSE Corning^®^ bench-top shaking incubator to perform the bench-scale washing of the contaminated swatches. All the samples were washed for 60 min at 40 °C and 300 RPM which was the maximum RPM available for the shaker bath. This high RPM would provide mechanical agitation during the washing process. The temperature of 40 °C was chosen in accordance with the NFPA 1851 standard. The fabric contamination and bench-scale washing processes are shown in Figure 1. After one hour of washing, contaminated water was drained, and samples were rinsed with 100 mL of clean water at room temperature for 10 min. In each batch, nine samples were washed using the bench-scale washer extractor. After washing, samples were placed in a rack to air dry for 24 h. Three replicates were used for each bench-scale washing condition.

##### Bench-Scale Washing without Presoaking

Bench-scale washing was performed as described in Section 2.2.3 on contaminated samples without any presoaking. The SDS of the detergent recommends using 6 oz of detergent for a 45 lb washing load. The weight of each fabric swatch was approximately 0.5 g. By scaling to the recommended amount, around 4.5 μL of detergent should be added for the bench-scale washing. Considering the additional 5 g of glass beads used for mechanical agitation, 50 μL of detergent was used during the washing process.

##### Effect of Presoaking Duration

Presoaking was performed using contaminated samples at a 90:10 water-to-detergent ratio according to the SDS of the detergent. However, presoaking was performed for varying durations (1 h, 3 h, and 12 h) before performing washing. The presoaking process took place in 250 mL Erlenmeyer flasks containing water and detergent. The combined volume of detergent and water in each flask was 100 mL. Following presoaking, the bench-scale washing process described in Section 2.2.3 was carried out. According to the SDS of the detergent, no additional detergent was added during the washing process after presoaking. The contaminated samples were washed in 250 mL Erlenmeyer flasks containing 5 g of glass beads and 100 mL of water without any additional detergent.

##### Effect of Detergent Concentration on Presoaked Sample Decontamination

To evaluate the impact of detergent concentration on decontamination, contaminated fabric swatches were presoaked in a 99:1 water-to-detergent ratio for 12 h, followed by water-only washing as described in Section 2.2.3. The cleaning efficacy of this approach was compared with that of samples presoaked in a 90:10 water-to-detergent ratio for 12 h. Furthermore, bench-scale washing was performed on another set of contaminated samples, using a 90:10 water-to-surfactant ratio without presoaking.

#### 2.2.4. Determination of Partition Coefficient (K) and Standard Affinity (Δμ^θ^)

In this experiment, the partition coefficient (K) describes the affinity of the PAH compounds for the outer-shell fabrics or the wash bath. So, the higher the K value, the more likely the compound partitions to the fabric as opposed to being desorbed into the wash bath. The partition coefficient is expressed as the ratio between the adsorbed mass of PAHs on the fabric to that transferred mass of PAHs in the washing bath at equilibrium after the washing process [35,36]. The unit of partition coefficient (K) is L/kg or mL/g. It is expressed using the following formula [37]:

(1) $$K=\frac{{\left[D\right]}_{f}}{{\left[D\right]}_{b}}$$

where [D]_f_ and [D]_b_ indicate the adsorbed mass of PAH per unit of fabric (“ng” PAH/“g” fabric) and adsorbed mass of PAH for every unit of washing bath solution (“ng” PAH/“mL” wash solution), respectively. The amount of PAH compound adsorbed per unit of fabric and the amount of PAH per unit of washing solution bath were determined from the cleaning efficacy, which provides the mass balance of PAH retained on the fabric and transferred to the solution after washing.

Standard affinity (Δμ^θ^) measures the driving force in the washing process. Standard affinity was calculated using the following formula [38]:

(2) $${\Delta \mu }^{\theta }=RTIn\frac{{\left[D\right]}_{f}}{{\left[D\right]}_{b}}$$

where R is the universal gas constant (in KJ K^−1^ mol^−1^), and T is the washing temperature (in Kelvin).

#### 2.2.5. PAH Measurement and Analysis Methods

To analyze the PAH concentrations on the fabrics, a Buchi Speed Extractor was used for the extraction process, followed by gas chromatography–mass spectrometry (GC-MS). The extraction and GC-MS analyses were carried out according to the method described by Hossain et al. [36]. Briefly, the extraction process was completed in a single cycle which consists of a one-minute heat-up period, a five-minute hold, and a two-minute discharge. Temperature and pressure were maintained at 100 °C and 100 bar, respectively. The extraction process took 18 min to complete. The extracted samples were analyzed using Agilent 7890B gas chromatographic system connected with Agilent 5977B mass spectrometer (Agilent, Santa Clara, CA, USA) equipped with electron ionization (EI). The oven temperature was initially set at 60 °C and increased to 200 °C at 30 °C/min followed by a one-minute hold. Subsequently, the temperature was increased to 300 °C at a rate of 10 °C/min and held for an additional five minutes. The total run time for each sample was 30 min.

The instrument was calibrated for each compound using six concentrations, ranging from 0.2 ng/μL to 8 ng/μL. The minimum R-square coefficient was 0.997 among all the calibration curves from each compound. To determine the limit of detection (LOD) and limit of quantitation (LOQ) for each compound, the lowest concentration (0.2 ng/μL) was run seven times consecutively. The following formulas were used to calculate the LOD and LOQ:

(3) $$LOD=\frac{3\sigma }{m}$$

(4) $$LOQ=\frac{10\sigma }{m}$$

Here, standard deviation (σ) is the peak area of seven consecutive runs and m is the slope of the calibration curve for each compound. The LOD and LOQ values for 16 targeted PAHs are shown in Table 1. LOQ/2 was used to determine PAH concentrations if no peaks were detected after washing.

#### 2.2.6. Determination of Cleaning Efficacy

Cleaning efficacy measures the concentration of contaminant removed by the washing process. It is calculated by the following formula:

(5) $$Cleaning\;efficacy=\frac{\left(Cc-Cm\right)-(Cw-Cp)}{\left(Cc-Cm\right)}\times 100$$

C_c_ = Original concentration of contaminant dosed on the fabric;

C_m_ = Amount of contaminant on the unwashed uncontaminated fabric;

C_w_ = Amount of contaminant on washed fabric;

C_p_ = Amount of contaminant on uncontaminated washed fabric.

If no peaks are detected in the GC-MS analysis of unwashed uncontaminated fabric or uncontaminated washed fabric, their contaminant concentrations are considered zero. Extraction efficiency was calculated and considered for each PAH compound to determine the removal efficacy for that compound when washing the fabrics. JMP Pro^®^ statistical software (15.2.0, SAS Institute Inc., Cary, NC, USA) was used to perform statistical analysis of removal efficacies of HMW PAHs after washing the fabrics.

## 3. Results

### 3.1. Removal of PAHs without Presoaking in Bench-Scale and Full-Scale Methods

**Figure 2:**
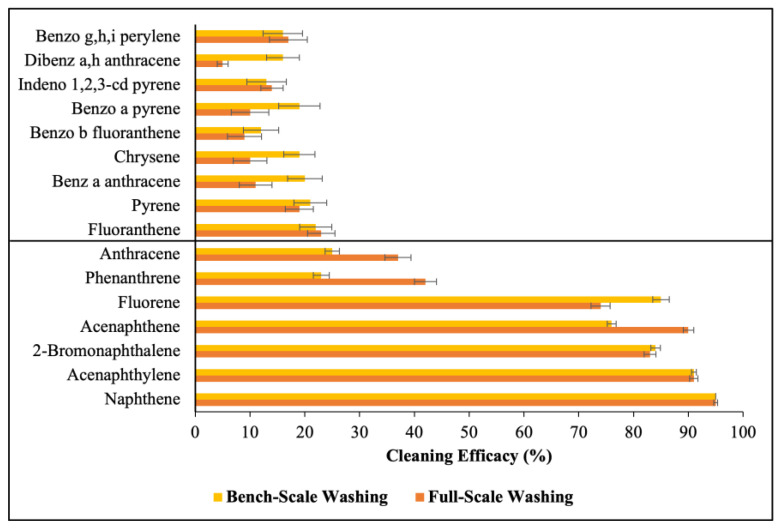
Cleaning efficacy of 16 PAHs for bench-scale and full-scale washing methods according to the SDS of the detergent; the first seven are LMW PAHs and the last nine are HMW PAHs; the error bar represents the standard error (SE) from the mean.

**Figure 3:**
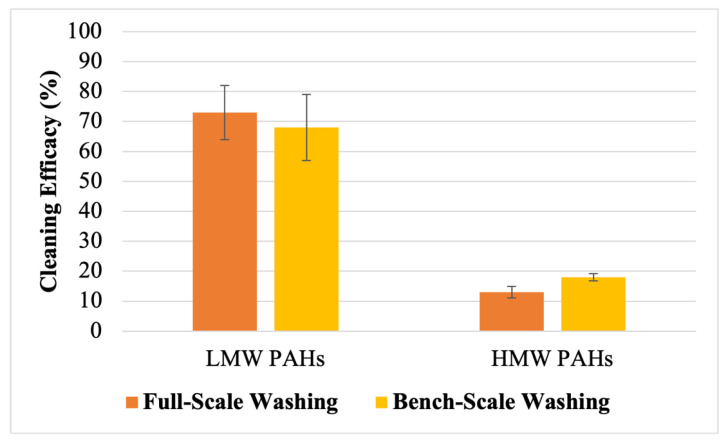
The average cleaning efficacy of Σ_7_ LMW and Σ_9_ HMW PAHs using bench-scale and full-scale washing methods according to the SDS of the detergent; the error bar represents the standard error from the mean.

The washing procedures were performed using both bench-scale and full-scale methods to determine the removal efficacy of 16 PAH compounds. This allowed for a comprehensive analysis of both methods regarding their removal efficacy for the PAH compounds. Figure 2 shows the removal efficacy against 16 PAHs using bench-scale and full-scale methods. The removal efficacy of LMW PAHs was significantly higher compared to HMW PAHs for both washing methods. Both the bench-scale and full-scale methods achieved a removal efficacy over 70% for Nap, Acy, 2-Br, Ace, and Fle. However, the removal efficacy decreased drastically for the HMW PAHs, with a removal efficacy less than 30% observed for all HMW PAHs for both washing methods. Figure 3 illustrates the average cleaning efficacy for Σ_7_ LMW and Σ_9_ HMW PAHs. The full-scale washing method removed 73 ± 9% of Σ_7_ LMW PAHs, while the bench-scale washing method removed 68 ± 11%. However, the removal efficacy for the HMW PAHs was slightly lower for the full-scale washing method compared to the bench-scale washing method, with cleaning efficacy rates of 13 ± 1.9% and 18 ± 1.2%, respectively, for Σ_9_ HMW PAHs.

In Table 2, the average partition coefficient and standard affinity are shown for Σ_7_ LMW PAHs and Σ_9_ HMW PAHs. For both the bench-scale and full-scale methods, the partition coefficient values are higher for Σ_9_ HMW PAHs compared to those of Σ_7_ LMW PAHs. For instance, in the bench-scale method, the partition coefficient values are 205.20 L/kg and 985.80 L/kg for Σ_7_ LMW PAHs and Σ_9_ HMW PAHs, respectively. In the full-scale method, these values are 111.50 and 1660.80 L/kg, respectively. A similar trend is observed for the standard affinity of the PAH compounds, with the standard affinity for Σ_9_ HMW PAHs being higher compared to that of Σ_7_ LMW PAHs for both washing methods. The partition coefficient and standard affinity values for all the individual compounds are shown in Table S1, which indicates that the HMW PAHs have higher partition coefficients and standard affinity values compared to the LMW PAHs. The results from both washing scales indicate that the low-molecular-weight compounds have a lower affinity for the fabric phase and a greater affinity for the detergent bath phase, leading to the desorption process proceeding further for those compounds. Conversely, the significantly higher K values for the high-molecular-weight compounds indicate that the adsorption process for the fabric is favored.

### 3.2. Effect of Presoaking Duration on PAH Removal

**Figure 4:**
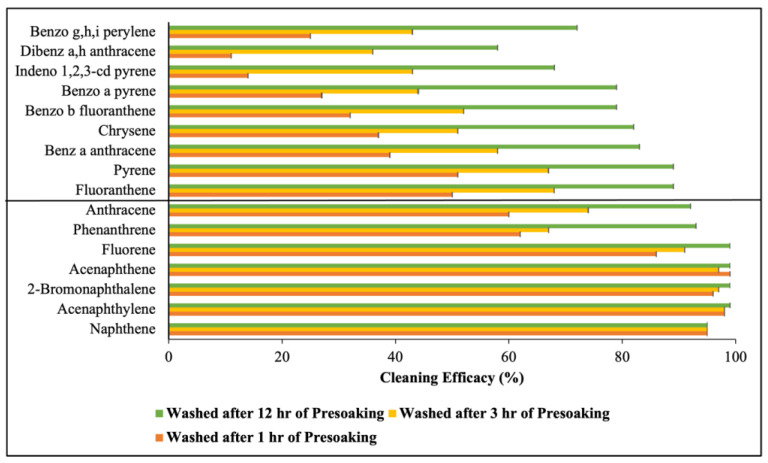
Cleaning efficacy of 16 PAHs after washing with different presoaking durations at a 90:10 water-to-detergent ratio; the first seven are LMW PAHs and the last nine are HMW PAHs; the error bar represents the standard error from the mean.

**Figure 5:**
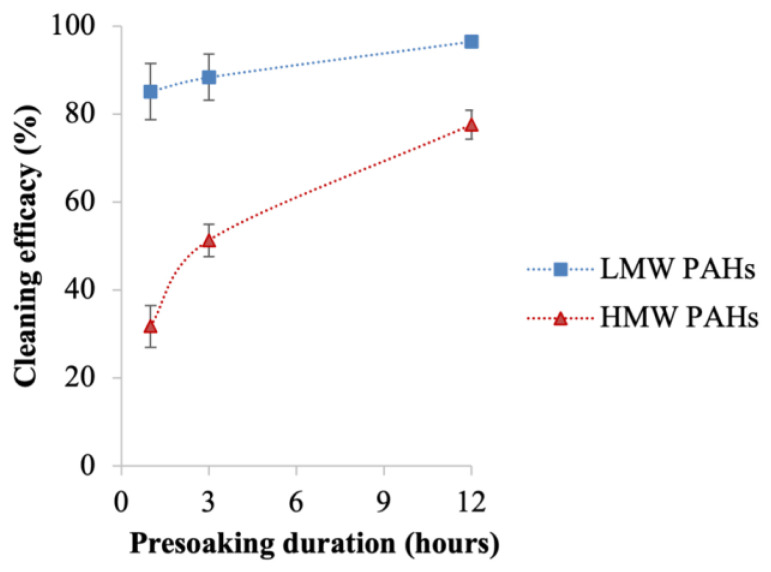
Average cleaning efficacy of Σ_7_ LMW and Σ_9_ HMW PAHs after washing fabrics with different presoaking durations at a 90:10 water-to-detergent ratio; the error bar represents the standard error from the mean.

Figure 4 illustrates the removal efficacy of the 16 PAHs with varying durations of presoaking before the bench-scale washing was conducted. As depicted, the removal efficacy for all 16 PAHs increases with longer presoaking durations. Within one hour of presoaking, a removal efficacy around 90% or higher was achieved for Nap, Acy, 2-Br, Ace, and Fle. Among the LMW PAHs, the lowest removal efficacy was observed for PHE (62 ± 0.01%) and An (60 ± 0.01%). The removal efficacy against some of the HMW PAHs was very low with the lowest being 11 ± 0.02% for D[ah]A. As the presoaking duration increased, the removal efficacy for all PAHs improved. With three hours of presoaking, individual LMW PAHs achieved a removal efficacy of 70% or higher, with that for PHE and An improving to around 70%. An improvement was also observed for the HMW PAHs, with the lowest removal efficacy being 36 ± 0.04% for D[ah]A. After 12 h of presoaking, a significant enhancement in removal efficacy was observed across all compounds. Notably, for the LMW PAHs, the lowest removal efficacy was observed for An (92 ± 0.004%), while for the HMW PAHs, the lowest was observed for D[ah]A (58 ± 0.005%). Figure 5 presents the average cleaning efficacy of Σ_7_ LMW and Σ_9_ HMW PAHs for various presoaking durations. It shows that one hour of presoaking removed 86 ± 6.4% of Σ_7_ LMW and only 32 ± 4.7% of Σ_9_ HMW PAHs. With three hours of presoaking, the removal efficacy improved to 89 ± 4.7% for Σ_7_ LMW and 51 ± 3.7% for Σ_9_ HMW PAHs. A substantial enhancement was observed after 12 h of presoaking, with removal efficacies reaching 97 ± 1.1% for Σ_7_ LMW PAHs and 78 ± 4.7% for Σ_9_ HMW PAHs. Paired Student’s *t*-tests indicated a significant difference (*p* < 0.05) in the removal efficacy of Σ_9_ HMW PAHs for different presoaking durations (shown in Figure S1).

**Figure 6:**
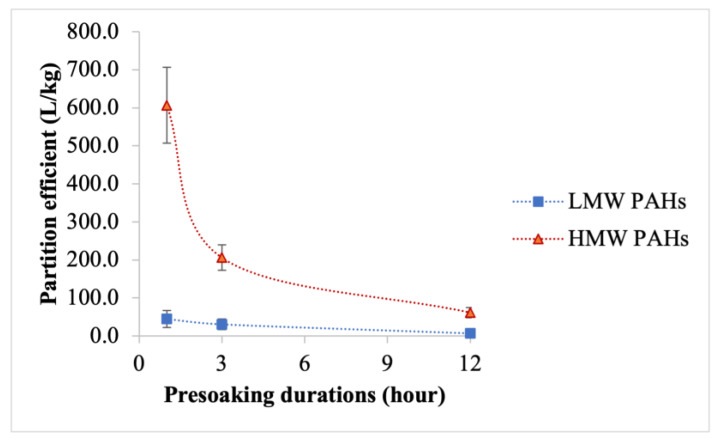
Average partition coefficient of Σ_7_ LMW PAHs and Σ_9_ HMW PAHs for different presoaking durations at a 90:10 water-to-detergent ratio; the error bar represents the standard error from the mean.

**Figure 7:**
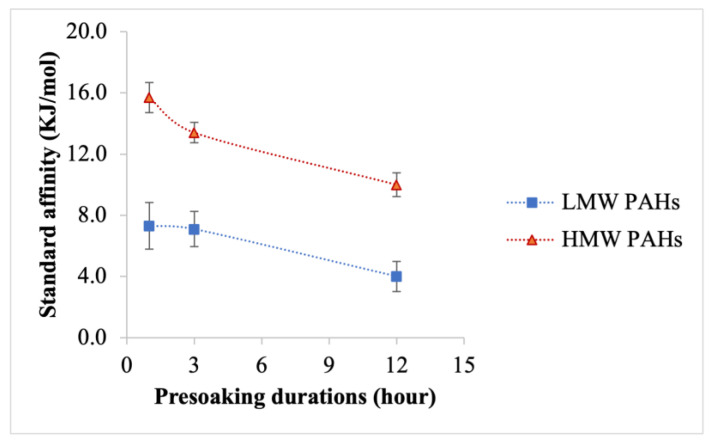
Average standard affinity of Σ_7_ LMW PAHs and Σ_9_ HMW PAHs for different presoaking durations at a 90:10 water-to-detergent ratio; the error bar represents the standard error from the mean.

Figure 6 and Figure 7 show the average partition coefficient and standard affinity, respectively, for Σ_7_ LMW PAHs and Σ_9_ HMW PAHs. An opposite trend is observed between these parameters and the removal efficacies of PAH compounds: as the removal efficacy increases, both the partition coefficient and standard affinity decrease. For Σ_7_ LMW PAHs, the partition coefficient values were highest after one hour of presoaking and lowest after 12 h of presoaking. Similarly, for Σ_9_ HMW PAHs, the partition coefficient peaked after one hour of presoaking and sharply declined after 12 h. The standard affinity values for PAH compounds followed the same pattern as that of the partition coefficient, with the lowest values observed after 12 h of presoaking. The partition coefficient and standard affinity were individually determined for all 16 PAH compounds with different presoaking durations (shown in Table S2). This demonstrated that the partition coefficient of each PAH compound decreases with longer presoaking durations, with the lowest values typically observed after 12 h of presoaking.

### 3.3. Effect of Detergent Concentration during Presoaking

**Figure 8:**
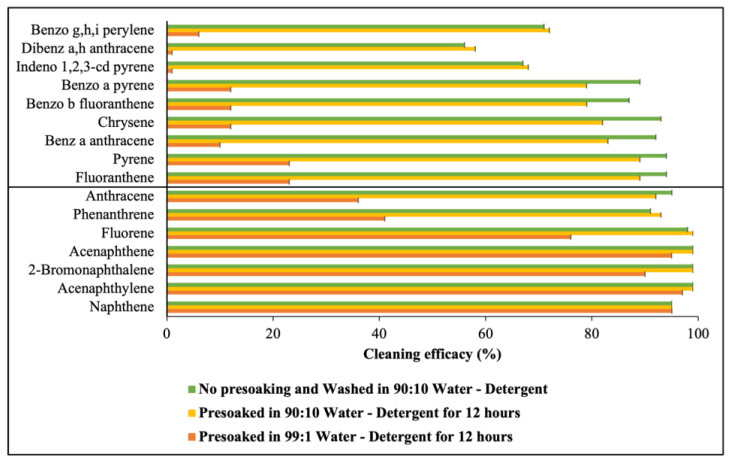
Cleaning efficacy of 16 PAHs with varying water-to-detergent ratios; the first seven are LMW PAHs and the last nine are HMW PAHs; the error bar represents the standard error from the mean.

**Figure 9:**
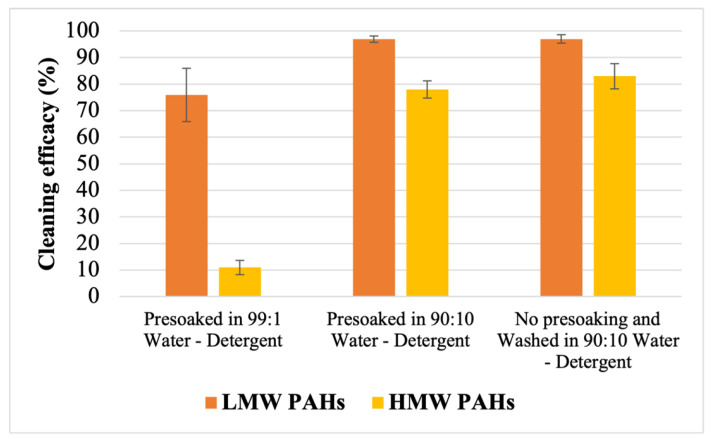
The average cleaning efficacy of Σ_7_ LMW and Σ_9_ HMW PAHs with varying water-to-detergent ratios; the error bar represents the standard error from the mean.

To evaluate the effect of detergent concentration during presoaking on the removal of PAHs, contaminated fabric swatches were presoaked for 12 h. They were presoaked separately using a 90:10 water-to-detergent ratio and a 99:1 water-to-detergent ratio (Figure 8). The results indicate that even after a 12 h presoaking period, the cleaning efficacy remained notably low, particularly for all the HMW PAHs, when presoaking was performed with a 99:1 water-to-detergent ratio. Figure 9 shows that only 11 ± 2.7% of the HMW PAHs were removed when presoaking was performed with a 99:1 water-to-detergent ratio. The removal efficacy was found to be lower than 10% against Ind, D[ah]A, and B[ghi]P. However, presoaking with a 90:10 water-to-detergent ratio increased the removal efficacy against all the PAH compounds, resulting in a removal efficacy of 78 ± 3.4% against Σ_9_ HMW PAHs. Furthermore, washing was conducted using a 90:10 water-to-detergent ratio directly in the washing bath without any presoaking. Figure 8 shows that the removal efficacy for the PAH compounds was nearly the same when the fabrics were washed without presoaking using a 90:10 water-to-detergent ratio in the bath compared to that of samples that were presoaked with the same ratio before washing without any additional detergent. Additionally, Figure 9 illustrates that in both scenarios, approximately 97% of the LMW PAHs and around 80% of the HMW PAHs were effectively removed. A paired Student’s *t*-test revealed no significant difference in cleaning efficacy for the HMW PAHs when washing was performed using a 90:10 water-to-detergent ratio after 12 h of presoaking compared to directly washing samples with the same ratio. However, a significant difference (*p* < 0.05) was observed when comparing washing with presoaking in a 99:1 water-to-detergent ratio to the other two approaches (shown in Figure S2).

In Table 3, the average partition coefficient and standard affinity are shown for Σ_7_ LMW PAHs and Σ_9_ HMW PAHs. When presoaking was performed with a 99:1 water-to-detergent ratio, the partition coefficient and standard affinity were found to be higher for both Σ_7_ LMW PAHs and Σ_9_ HMW PAHs compared to the other washing methods, indicating a stronger affinity for adsorption to the fabrics. However, when comparing washing with a 90:10 water-to-detergent ratio, whether with or without presoaking, the partition coefficient and standard affinity were nearly the same for Σ_7_ LMW PAHs and Σ_9_ HMW PAHs in both scenarios. The partition coefficient and standard affinity values for all the individual compounds with varying water-to-detergent ratios are shown in Table S3.

## 4. Discussion

### 4.1. Removal of PAHs without Presoaking in Bench-Scale and Full-Scale Methods

The targeted 16 PAH compounds analyzed in this study have between two and six condensed or fused aromatic rings in their structure. With the increase in the fused rings in their structure, the molecular weight and octanol-water partition coefficient (*K*_OW_) value of the PAHs increase. Among various physico-chemical parameters, the *K*_OW_ value is the key parameter that defines the distribution of PAHs between the aqueous phase and organic phases, thus indicating their hydrophobicity [39]. The *K*_OW_ values of the LMW PAHs and the HMW PAHs in this study range from 3.29 to 4.45 and 4.9 to 6.5, respectively. The lower *K*_OW_ values of the LMW PAHs indicate their higher solubility in water, which makes them more readily removable during washing processes [31]. Consequently, both the bench-scale and full-scale washing methods exhibited a greater efficacy in removing the LMW PAHs, with removal rates of approximately 68% and 73%, respectively. This indicates that the routine laundering process following NFPA guidelines and manufacturer-recommend detergent can effectively remove LMW PAHs from contaminated turnout gear. However, the laundering process proves inadequate in removing high-molecular-weight (HMW) PAHs, with full-scale washing achieving only a 13% removal of Σ_9_ HMW PAHs from the turnout gear fabrics. High-molecular-weight PAHs exhibit a higher affinity to fabrics due to their higher *K_OW_* values and lower water solubility [40]. Therefore, these compounds are not effectively removed from fabrics using the recommended concentration of the detergent and the laundering process evaluated in this study. This trend is also consistent for bench-scale washing in which only 18% of Σ_9_ HMW PAHs were removed. Compounds containing five or six aromatic rings, due to their higher *K_OW_* values, exhibited the lowest removal efficacy. These findings highlight that PAH removal during the washing process largely depends on the physico-chemical properties of PAHs and indicates that the bench-scale method can adequately replicate full-scale washing.

The removal of PAHs from fabrics exhibits a reverse correlation with their partition coefficient and standard affinity in solution. Consequently, LMW PAHs typically have lower partition coefficients and standard affinities compared to HMW PAHs, which explains their higher removal efficacy from fabrics after washing. The partition coefficient is determined by the ratio of PAHs adsorbed on the fabric to those transferred to the washing bath. This means that as the removal efficacy for a PAH compound increases, the adsorbed mass of the PAH compound per unit of the fabric decreases, while the mass of the PAH compound in the washing solution increases. Consequently, with the increase in the removal efficacy of a PAH compound, its partition coefficient decreases. A lower partition coefficient indicates that a smaller amount of the PAH compound is adsorbed on the fabric, with a larger proportion transferring to the washing bath. In contrast, HMW PAHs have higher partition coefficients because a higher concentration of these compounds remains adsorbed on the fabrics compared to what is transferred to the washing bath. The standard affinity determines the tendency of PAH molecules to move from their standard state in the fiber to their standard state in the solution [41]. Higher standard affinity values for HMW PAHs indicate a lower tendency for these compounds to desorb from the fabric into the wash bath compared to LMW PAHs. Consequently, the standard affinity values are higher for Σ_9_ HMW PAHs compared to Σ_7_ LMW PAHs across both washing methods, reflecting their greater affinity for fabric surfaces and lower removal rates during laundering.

### 4.2. Effect of Presoaking Duration on PAH Removal

Presoaking was performed for varying durations (1 h, 3 h, and 12 h) before washing to understand the impact of longer overnight presoaking (12 h) versus shorter durations (one hour or three hours) on PAH removal. These specific durations were chosen considering that overnight presoaking (12 h) might not always be feasible. Performing presoaking for one hour and three hours would be helpful to determine if shorter presoaking durations could offer significant decontamination benefits. The results indicate that the effectiveness of PAH compound removal can significantly depend on the presoaking duration before washing at a constant detergent concentration. Within an hour of presoaking, approximately 85% of Σ_7_ LMW PAHs are successfully removed from the fabrics. However, due to the greater hydrophobicity of HMW PAHs, a low portion of the PAHs were desorbed from the fabric to the solution after washing them and only 32% of Σ_9_ HMW PAHs were eliminated after one hour of presoaking. This indicates that the adsorbed mass of PAHs per unit of fabric (ng/g) was highest after one hour of presoaking, resulting in the highest partition coefficient and standard affinity values for both the low- and high-molecular-weight PAHs. As the presoaking durations increase, the detergent in the bath has more time to interact with PAH molecules, facilitating increased desorption from the fabrics. Therefore, the removal efficacy improves significantly after 3 or 12 h of presoaking. For instance, three hours of presoaking removes around 89% of Σ_7_ LMW PAHs and 51% of Σ_9_ HMW PAHs from the fabrics. Compared to the 18% removal efficacy achieved through bench-scale washing without presoaking, the 51% removal after three hours of presoaking represents a notable 2.8-fold increase. Since a substantial amount of LMW PAHs are already desorbed from the fabric within one hour of presoaking, further increases in presoaking duration do not significantly impact the desorption of the remaining LMW PAHs from the fabrics. Therefore, the removal efficacy for Σ_7_ LMW PAHs does not improve as markedly as it does for Σ_9_ HMW PAHs after three hours of presoaking. However, a further investigation with a 12 h presoaking period reveals an enhanced removal efficacy across all PAH compounds. The removal efficacy reaches 97% and 78% for low- and high-molecular-weight PAHs, respectively, indicating that an overnight presoaking can effectively remove both LMW and HMW PAHs from turnout gear. After the 12 h presoaking period, the partition coefficient and standard affinity for the PAH compounds are found to be the lowest, indicating that minimal amounts of PAHs remain adsorbed on the fabrics after washing, with most of the PAHs transferring to the washing bath. This finding indicates that the desorption process evaluated in this study requires more than three hours to reach a kinetic steady-state condition. However, considering practical constraints such as gear availability and turnaround time, a 12 h presoaking period may prove impractical. Thus, firefighters may opt for shorter presoaking durations to achieve a higher cleaning performance compared to washing without presoaking, balancing effectiveness with operational efficiency.

### 4.3. Impact of Detergent Concentration during Presoaking

Surfactants play a crucial role in removing hydrophobic organic compounds like PAHs from contaminated soil by enhancing the solubility of PAHs [42,43,44]. Surfactants desorb PAHs from contaminated fabrics by incorporating PAH molecules into the aqueous phase and portioning them into the hydrophobic cores of surfactant micelles [43,45]. To determine the role of surfactant concentration during presoaking, a 12 h presoaking period was tested using different water-to-detergent ratios, and the removal efficacy for 16 PAHs was evaluated. For presoaking, a 90:10 water-to-detergent ratio was used based on the SDS of the detergent. Additionally, a lower detergent ratio (a 99:1 water-to-detergent ratio) was selected randomly to investigate the effectiveness of a lower detergent concentration in desorbing PAHs from fabrics. The results indicate that presoaking contaminated fabrics in a 99:1 water-to-detergent solution fails to effectively remove persistent PAHs. While 76% of Σ_7_ LMW PAHs were eliminated, only 11% of Σ_9_ HMW PAHs were removed after washing. The partition coefficient and standard affinity for the PAH compounds were found to be the highest due to the minimal desorption of the PAHs after washing. If the surfactant concentration is below the critical micelle concentration (CMC), the surfactant will create a monolayer on the fabric’s surface, which is ineffective in desorbing PAHs from the fabric. However, when the concentration exceeds the CMC, its ability to desorb PAHs from the fabric to the solution improves significantly. For highly hydrophobic compounds like PAHs, the excess surfactant beyond the CMC leads to an increased micelle volume in the bulk solution, which can desorb a higher portion of PAHs from the fabric to the solution [45]. Therefore, presoaking for the same duration but with a 90:10 water-to-detergent ratio before washing resulted in the removal of 97% of Σ_7_ LMW PAHs and 78% of Σ_9_HMW PAHs. The partition coefficient and standard affinity for the PAH compounds were decreased significantly due to the sharp decrease in the adsorbed mass of the PAHs on the fabrics after washing. This proves the pivotal role of detergent volume or water-to-detergent ratios during presoaking in PAH removal from turnout gear. Given the significance of surfactant volume, washing was also conducted without presoaking, directly employing a 90:10 water-to-detergent ratio in the washing bath. A pomparative analysis shows a similar removal efficacy for 16 PAHs when presoaking with a 90:10 ratio and direct washing with the same ratio. Specifically, washing with a 90:10 ratio directly achieved a 97% removal of the LMW PAHs and an 83% removal of the HMW PAHs. The reason for obtaining such a high and similar removal efficacy for the 90:10 water-to-detergent ratio is that 10 mL of detergent could effectively desorb all the persistent hydrophobic PAHs from the fabric [36]. This indicates that using a higher detergent concentration like a 90:10 water-to-detergent ratio directly in a laundering machine can desorb PAHs from the fabric even without presoaking it. Therefore, almost the same cleaning efficacy was achieved irrespective of presoaking, using a 90:10 water-to-detergent ratio during washing. Consequently, the partition coefficient and standard affinity for LMW PAHs and HMW PAHs were found to be almost the same for both washing approaches. However, considering the volume of water used in the wash extractor during the washing process, using such a ratio without presoaking would necessitate a higher detergent volume in the washing machine. Conversely, presoaking using a 90:10 ratio before washing offers a simpler and more feasible approach with reduced detergent consumption. However, further investigations are required to determine whether presoaking with a higher surfactant concentration (a 90:10 water-to-detergent ratio) for an extended duration has any negative impact on the performance of the outer-shell layer of the turnout gear.

### 4.4. Limitations

The detergent used in this experiment contains a non-ionic surfactant, and the findings related to removal efficacies, partition coefficients, and standard affinity for PAH compounds are specific to this detergent. Replicating this study with a different detergent would likely yield different results depending on the detergent formulation, though similar trends are expected. Additionally, this research focused exclusively on PAH compounds. However, it is possible that the methodology could be applied to other classes of compounds, such as phenols, phthalates, per- and polyfluoroalkyl substances (PFAS), and flame retardants, although further testing would be necessary to confirm this. Overall, this experiment could facilitate more targeted testing with a smaller range of compounds.

## 5. Conclusions

The primary objective of turnout gear is to protect firefighters from heat, flames, and chemical hazards. However, over time, turnout gear can become contaminated with toxic substances, including persistent hydrophobic organic compounds like PAHs. The outer-shell layer of turnout gear accumulates the highest concentration of contaminants, as soot particles containing these chemicals deposit on the outer part first. Contaminated gear poses severe health risks to firefighters by acting as a source of repetitive exposure to carcinogenic contaminants and can also compromise the protective performance of the gear. Routine laundering is not sufficient to effectively remove these persistent compounds. Maintaining clean turnout gear by removing persistent chemicals is crucial for the health and safety of firefighters. To assess the effect of presoaking duration, a representative fabric was presoaked in a 90:10 water-to-detergent mixture, as recommended by the detergent’s SDS, for different durations. The findings indicate that the removal of PAHs is significantly influenced by the duration of presoaking. Additionally, the volume of surfactant used in the water–surfactant mixture during presoaking plays a crucial role in the decontamination process. Furthermore, adding detergent directly into the washer/extractor while maintaining a 90:10 water-to-detergent ratio can achieve the same removal performance as presoaking for 12 h before washing, under the assumption that the washing equipment can handle that ratio. Our research shows that the removal or desorption of PAHs from contaminated outer-shell fabrics is influenced by the partition coefficient and standard affinity of PAHs in the solution. These properties explain how PAHs can be released from fabric surfaces into the washing solution, underscoring their critical role in the determination of the removal efficacies of PAHs. Overall, this study provides essential insights for firefighters and the firefighting industry on optimizing presoaking practices to effectively clean turnout gear, thereby enhancing the safety of firefighters by reducing chemical exposure.

## Figures and Tables

**Table 1:** Chemical and physical properties of 16 targeted PAHs (adapted from [31]).

PAH Compound	Molecular Weight (g/mol)	Number of Rings	Boiling Point (°C)	Octanol-Water Partitioning Coefficient (Log *K_OW_*)	Solubility in Water (mg/L)	LOD * (ng/μL)	LOQ * (ng/μL)
Naphthalene (Nap)	128.17	2	218	3.29	31.000	0.10	0.33
Acenaphthylene (Acy)	152.20	3	280	4.07	3.800	0.03	0.10
2-Bromo naphthalene (2-Br)	207.00	2	281	No data	3.400	0.03	0.09
Acenaphthene (Ace)	154.21	3	279	3.98	0.045	0.03	0.12
Fluorene (Fle)	166.22	3	295	4.18	1.900	0.03	0.11
Phenanthrene (PHE)	178.23	3	340	4.45	1.100	0.04	0.12
Anthracene (An)	178.23	3	340	4.45	0.045	0.03	0.10
Fluoranthene (Fla)	202.25	4	404	4.90	0.260	0.03	0.09
Pyrene (Py)	202.26	4	400	4.88	0.132	0.02	0.07
Benz[a]anthracene (B[a]A)	228.29	4	438	5.61	0.011	0.03	0.11
Chrysene (Chr)	228.29	4	448	5.90	0.001	0.02	0.05
Benzo[b]fluoranthene (B[b]F)	252.32	5	481	6.04	0.001	0.03	0.09
Benzo[a]pyrene (B[a]P)	252.32	5	495	6.06	0.004	0.03	0.09
Indeno[1,2,3-cd]pyrene (Ind)	276.33	6	530	6.58	0.062	0.03	0.10
Dibenz[a,h]anthracene (D[ah]A)	278.35	5	524	6.84	0.001	0.06	0.19
Benzo[g,h,i]perylene (B[ghi]P)	276.33	6	550	6.50	0.0002	0.07	0.22

* Limit of detection (LOD) and limit of quantitation (LOQ) values are provided for GC/MS analysis.

**Table 2:** Partition coefficient (K), and Standard affinity (Δμ^θ^) of Σ_7_ LMW PAHs and Σ_9_ HMW PAHs for both washing methods according to the SDS of the detergent (range = mean ± SE (standard error)).

PAH Compounds	K (L/kg)		(Δμ^θ^); KJ/mol	
	Bench-Scale Washing	Full-Scale Washing	Bench-Scale Washing	Full-Scale Washing
LMW PAHs	205.2 ± 109.4	111.5 ± 57.1	11.0 ± 1.6	10.4 ± 1.3
HMW PAHs	985.8 ± 144.9	1660.8 ± 344	17.9 ± 0.9	18.9 ± 1.3

**Table 3:** Partition coefficient (K) and Standard affinity (Δμ^θ^) of Σ_7_ LMW PAHs and Σ_9_ HMW PAHs for varying water-to-detergent ratios (range = mean ± SE).

PAH Compounds	K (L/kg)			(Δμ^θ^); KJ/mol		
	Presoaked in 99:1 Water—Detergent for 12 h	Presoaked in 90:10 Water—Detergent for 12 h	No Presoaking and Washed in 90:10 Water—Detergent	Presoaked in 99:1 Water—Detergent for 12 h	Presoaked in 90:10 Water—Detergent for 12 h	No Presoaking and Washed in 90:10 Water—Detergent
LMW PAHs	108.0 ± 85.7	7.2 ± 3.9	7.2 ± 3.8	10.8 ± 2.6	5.1 ± 1.6	5.1 ± 1.4
HMW PAHs	5585.0 ± 2696.0	61.9 ± 12.9	49.9 ± 17.1	20.3 ± 1.1	10.3 ± 0.5	11.4 ± 0.8

## Data Availability

The data presented in this study are available on request from the corresponding author.

## Supplementary Materials

The following supporting information can be downloaded at: https://www.mdpi.com/article/10.3390/toxics12080544/s1, Figure S1: Each pair Student’s *t*-test for the removal efficacy of 9 ΣHMW PAHs after washing with different presoaking durations; Figure S2: Each pair Student’s *t*-test for the removal efficacy of 9 ΣHMW PAHs after washing with varying water-to-detergent ratios; Table S1: Partition coefficient, and standard affinity of PAH compounds for both washing methods [range = mean ± SE]; Table S2: Partition coefficient and standard affinity of PAH compounds for different durations of presoaking [range = mean ± SE]; Table S3: Partition coefficient and standard affinity of PAH compounds for varying water-to-detergent ratios [range = mean ± SE].
