# Supplementary material for: Assessing the Impact of Pre-Soaking to Enhance Laundering Efficacy of Firefighter Turnout Gear
Source: Toxics. 2024 Jul 27;12(8):544. doi: 10.3390/toxics12080544 (PMC11358897; doi:10.3390/toxics12080544)
Supplement: Supplementary file 1 [file toxics-12-00544-s001.zip › Supplementary Tables copy.pdf]

Table S1

PAH Compounds	K (L/kg)		(Δμθ); KJ/mol	
	Bench-Scale washing	Full-Scale Washing	Bench-Scale washing	Full-Scale Washing
Nap	10.5±0	10.5±0	6.1±0	6.1±0
Acy	19.8±1.1	19.8±0.6	51.5±2.8	51.5±1.6
2-Br	38.1±2.5	41.0±1.4	9.5±0.1	9.7±0.1
Ace	63.2±2.8	22.2±0.8	10.8±0.1	8.1±0.1
Fle	35.3±9.7	70.3±1.6	9.3±0.2	11.1±0.1
PHE	669.6±57.1	276.2±10.4	16.9±0.2	14.6±0.1
An	600.0±40.9	340.5±15.9	16.6±0.2	15.2±0.1
Fla	709.1±131.9	669.6±76.6	17.1±0.5	16.9±0.3
Py	752.4±145.0	852.6±124.4	17.2±0.5	17.6±0.3
B[a]A	800.0±181.0	1618.2±482.2	17.4±0.5	19.2±0.7
Chr	852.6±182.6	1800.0±757.6	17.6±1.7	19.5±1.7
B[b]F	1466.7±699.0	2022.2±1356.1	19.0±0.9	19.8±1.1
B[a]P	852.6±281.7	1800.0±1000.0	17.6±0.7	19.5±1.1
**Ind**	1338.5±567.9	1228.6±566.0	18.7±0.9	18.5±0.6
**D[ah]A**	1050.0±825.3	3800.0±0	18.1±1.4	21.4±0
**B[ghi]P**	1050.0±433.7	976.5±300.0	18.1±0.8	17.9±0.8

Table S2

PAH Compounds	K (L/kg)			(Δμθ); KJ/mol		
	**1 hour**	3 hour	12 hour	1 hour	3 hour	12 hour
Nap	10.5±0	10.5±0	10.5±0	6.1±0	6.1±0	6.1±0
Acy	4.1±1.5	4.1±0.9	2.0±0.4	3.4±0.9	3.2±0.6	2.14±0.5
2-Br	8.3±2	6.2±1.4	2.0±0.1	5.5±0.7	4.7±0.6	1.8±0.2
Ace	2.0±0.8	6.2±0.9	2.0±0	1.8±0.7	4.7±0.4	1.8±0
Fle	32.6±3.0	19.8±3.5	2.0±0	9.1±0.2	7.8±0.5	1.8±0
PHE	122.6±5.0	98.5±4.7	15.1±1.0	12.5±0.1	11.9±0.1	7.10±0.2
An	133.3±6.3	70.3±8.1	17.4±1.1	12.7±0.2	11.1±0.3	7.4±0.2

Fla	200.0±11.0	94.1±8.6	24.7±1.8	13.8±0.1	11.8±0.2	8.3±0.2
Py	192.2±10.5	98.5±9.0	24.7±1.0	13.7±0.1	11.9±0.2	8.3±0.1
B[a]A	312.8±42.4	144.8±11.3	41.0±1.4	15.0±0.3	12.9±0.2	9.7±0.1
Chr	340.5±25.1	192.2±15.1	43.9±1.0	12.5±1.7	11.9±1.7	7.1±1.7
B[b]F	425.0±48.7	184.6±9.5	53.2±3.2	15.7±0.3	13.6±0.1	10.3±0.2
B[a]P	540.7±152.7	254.5±57.4	53.2±8.0	16.4±0.6	14.4±0.6	10.3±0.4
Ind	1228.6±278.0	265.1±22.9	94.1±2.5	18.5±0.5	14.5±0.2	11.8±0.1
D[ah]A	1618.2±243.7	355.6±40.2	144.8±3.22	19.2±0.4	15.3±0.3	12.9±0.1
B[ghi]P	600.0±76.2	265.1±29.0	77.8±6.2	16.6±0.3	14.5±0.2	11.3±0.2

Table S3

PAH Compounds	K (L/kg)			(Δμθ); KJ/mol		
	Presoaked in 99:1 Water - Detergent for 12 hours	Presoaked in 90:10 Water - Detergent for 12 hours	No presoaking and Washed in 90:10 Water - Detergent	Presoaked in 99:1 Water - Detergent for 12 hours	Presoaked in 90:10 Water - Detergent for 12 hours	No presoaking and Washed in 90:10 Water - Detergent
Nap	10.5±0	10.5±0	10.5±0	6.1±0	6.1±0	6.13±0
Acy	6.1±2.7	2.0±0.4	2.0±0.2	3.7±0.1	2.1±0.5	2.41±0.2
2-Br	22.2±2.7	2.0±0.1	2.0±0	8.0±0.3	1.8±0.2	1.83±0
Ace	10.5±1.3	2.0±0	2.0±0	6.1±0.4	1.8±0	1.83±0
Fle	63.1±3.3	2.0±0	4.1±0.3	10.7±0.1	1.8±0	3.6±0.2
PHE	287.8±20.4	15.1±1.0	19.8±4.8	14.7±0.2	7.1±0.2	7.7±0.8
An	355.5±28.9	17.4±1.1	10.5±0.2	15.2±0.2	7.4±0.2	6.1±0.1
Fla	669.5±82.2	24.7±1.8	12.8±0.6	16.9±0.3	8.3±0.2	6.6±0.1
Py	669.5±82.0	24.7±1.0	12.8±0.6	16.9±0.3	8.3±0.1	6.6±0.1
B[a]A	1800.0±600.0	41.0±1.4	17.3±0.7	19.5±0.7	9.7±0.1	7.4±0.1
Chr	1466.6±354.0	43.9±1.0	15.0±0.6	18.9±1.7	7.1±1.7	7.1±1.7
B[b]F	1466.6±389.0	53.2±3.2	29.8±1.7	18.9±0.6	10.3±0.2	8.8±0.1
B[a]P	1466.6±542.1	53.2±8.0	24.7±0.8	18.9±0.7	10.3±0.4	8.3±0.1
Ind	19800.0±0	94.1±2.5	98.5±5.3	25.7±0	11.8±0.1	11.9±0.1
D[ah]A	19800.0±0	144.8±3.2	157.1±6.8	25.7±0	12.9±0.1	13.1±0.1
B[ghi]P	19800.0±0	77.8±6.2	81.6±6.3	20.9±0	11.3±0.2	11.4±0.2
