# Supplementary figures and images for: Assessing the Impact of Pre-Soaking to Enhance Laundering Efficacy of Firefighter Turnout Gear
Source: Toxics. 2024 Jul 27;12(8):544. doi: 10.3390/toxics12080544 (PMC11358897; doi:10.3390/toxics12080544)

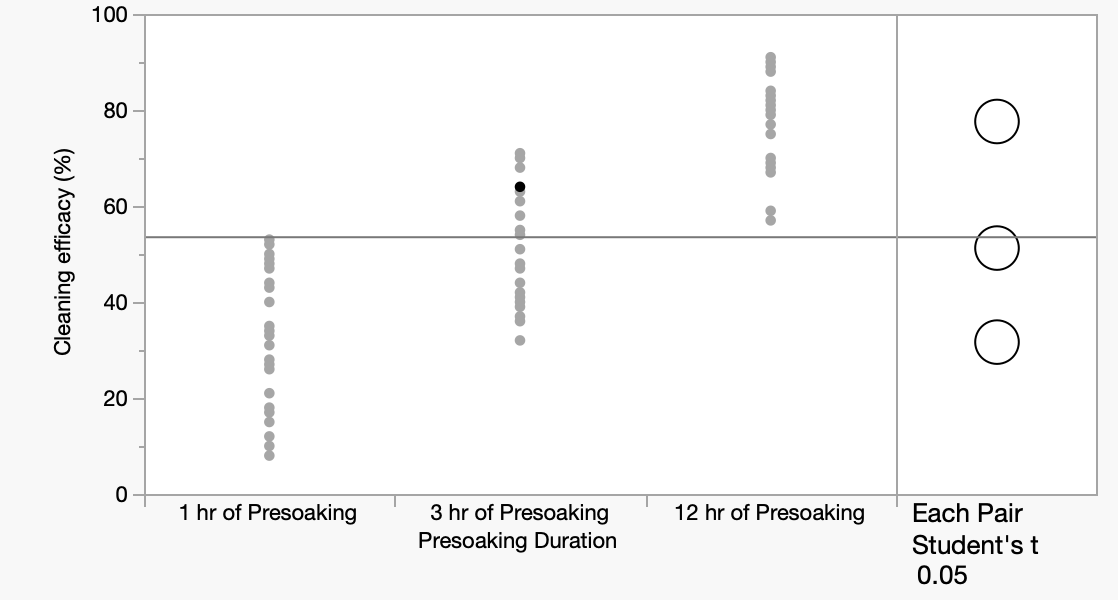

Supplement: Supplementary file 1 [file toxics-12-00544-s001.zip › Figure S1.png]

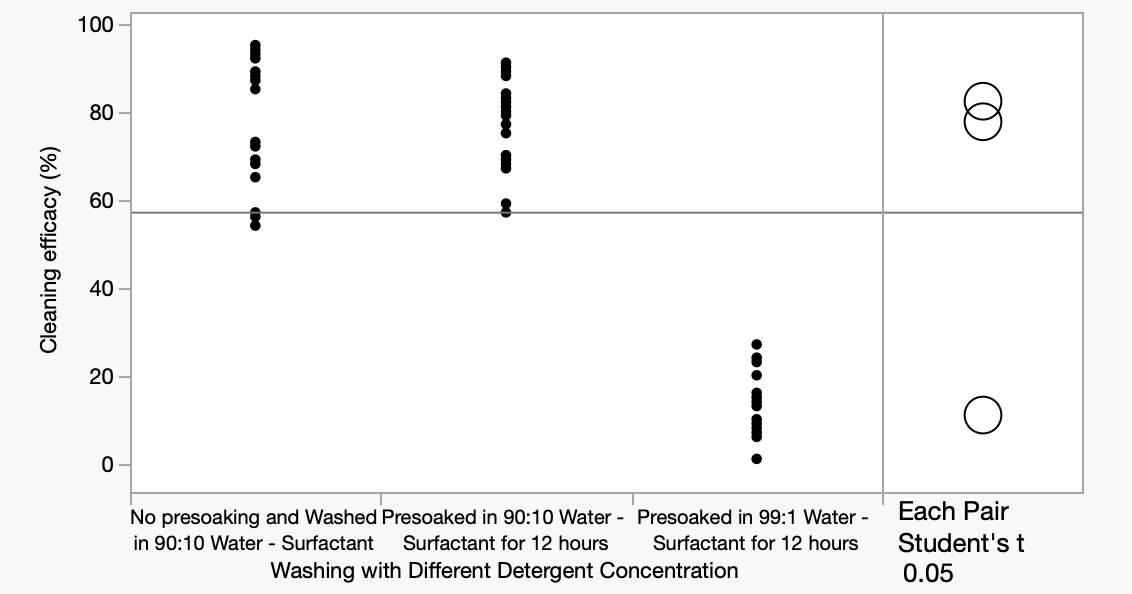

Supplement: Supplementary file 1 [file toxics-12-00544-s001.zip › Figure S2.png]
